# Cognitive Fusion, Ruminative Response Style and Depressive Spectrum Symptoms in a Sample of University Students

**DOI:** 10.3390/life13030803

**Published:** 2023-03-15

**Authors:** Mario Miniati, Sara Busia, Ciro Conversano, Graziella Orrù, Rebecca Ciacchini, Viarda Cosentino, Donatella Marazziti, Angelo Gemignani, Laura Palagini

**Affiliations:** 1 Department of Clinical and Experimental Medicine, University of Pisa, 56126 Pisa, Italy; viardacos@gmail.com (V.C.); dmarazzi@med.unipi.it (D.M.); lpalagini@gmail.com (L.P.); 2 Department of Surgical Medical and Molecular Pathology, Critical Care and Care Medicine, University of Pisa, 56126 Pisa, Italy; s.busia@studenti.unipi.it (S.B.); ciro.conversano@unipi.it (C.C.); graziella.orru@unipi.it (G.O.); rebecca.ciacchini@med.unipi.it (R.C.); angelo.gemignani@unipi.it (A.G.)

**Keywords:** university students, depression, ruminative response, cognitive fusion, psychological inflexibility

## Abstract

Psychological inflexibility is related to depressive symptoms through the ‘ruminative response style’ (RR) and ‘cognitive fusion’ (CF). We aimed at exploring whether university students were more exposed to CF, RR and depressive symptoms because of their intellectual performance than non-university students of the same age. We compared university students (US) (*n* = 105) vs. non-university students (NUS) (*n* = 76) through online administration of the ‘Cognitive Fusion Questionnaire’ (CFQ-7), the ‘Depression-Zung Self-Assessment Scale’ (ZSDS) and the ‘Perseverative Thinking Questionnaire’ (PTQ) (study protocol #0077818/2022, approved by the Ethical Committee of the University of Pisa, Italy). University students scored significantly higher than non-university students in the CFQ-7 Total Score (27.5 ± 9.4 vs. 24.4 ± 9.5; *p* = 0.040), ZSDS Total Score (41.1 ± 7.7 vs. 39.0 ± 7.3; *p* = 0.031), PTQ Total Score (26.1 ± 13.1 vs. 21.8 ± 13.9; *p* = 0.029), PTQ ‘Repetitiveness’ (5.3 ± 2.8 vs. 4.5 ± 2.9; *p* = 0.034), ‘Intrusiveness’ (5.8 ± 3.0 vs. 4.8 ± 3.1; *p* = 0.046) and ‘Repetitive Negative Thinking capturing mental resources’ (5.0 ± 3.1 vs. 4.0 ± 3.0; *p* = 0.013) (MANOVA analysis). In a binary logistic regression analysis of US (with ZSDS scores < 44 vs. ≥44 as the dependent variable, and PTQ Total Score and dimensions, CFQ-7 Total Score, age and gender as the covariates), PTQ Total Score predicted the more severe depressive symptomatology (OR = 1.44, 95% CI: 1.017–2.039; *p* = 0.040). We believe that RR and CF should be specifically targeted through psychoeducational/psychotherapeutic interventions in university students.

## 1. Introduction

Depression, even in its most attenuated forms, is spreading and interfering with the quality of life (QoL) in community samples [1,2]. Several studies indicated that depressive disorders might have onset during emerging adulthood, an evolutionary phase characterized by multiple role transitions, with environmental and social demands (such as the role transition to university), favoring the occurrence of depressive signs and symptoms [3,4,5,6]. The specific weight of psychological dimensions, which may favor the onset of depressive spectrum conditions (namely the broad area of psychological/psychiatric phenomenology relating to depression and including signs, isolated symptoms, symptom clusters and behavioral patterns, prodromes and precursors) [7,8,9] in the general population samples of young adults is still under debate [10,11]. Among the psychological dimensions related to the occurrence of depressive spectrum symptoms, ‘psychological inflexibility’ has recently been considered noteworthy, especially in emerging adulthood [12,13,14]. Psychological inflexibility is characterized by a loss of contact with current environmental and psychological experiences and by the use of an unchanged and stereotyped repertoire of emotional and cognitive responses [10,15]. This dimension is linked to the ‘ruminative response style’ (RR), namely to a cognitive response style, which involves passively brooding about one’s mood and, to some extent, to the occurrence of depressive signs and symptoms throughout the process of ‘cognitive fusion’ (CF). Cognitive fusion (CF) (namely the tendency to remain ‘trapped in thoughts’ and to consider them as if they were literally and objectively true) leads to excessive adhesion to one’s own thoughts and to the regulation of behavior mainly guided by cognition and other internal experiences rather ‘than by the direct experience with the world’ [11]. Rigid patterns of CF have been proposed as a risk factor for various forms of psychopathology, including anxiety and depressive disorders [16,17,18,19,20,21,22]. Cognitive fusion (CF) can exacerbate subjective suffering (e.g., sadness, anxiety, anger, guilt), may narrow behavioral repertoires and hinder effective actions for a ‘meaningful life’ [18]. This condition is characterized by negative self-referential thoughts, such as ‘I am inadequate’, which can elicit unpleasant mood states (e.g., sadness) and may make effective actions less probable, leading to the use of unhelpful avoidance strategies to reduce discomfort, such as worrying, thought suppression or ‘ruminative responses’ (RR) [23].

According to a number of recent observations, university students seem to be a special population at high risk of psychological strain related to depression, especially over the last 3 years, characterized by the compulsions of social lockdown and social distancing due to the COVID-19 pandemic [24]. High rates of psychological distress (and even of suicidal ideation) were described among university students who experienced increased levels of stress, anxiety and depression [25,26,27]. Several factors were considered as related to depressive signs and symptoms, such as the academic workload [28], performance pressure [29] and a pervasive sense of uncertainty regarding future career prospects [30]. Yet, less attention has been paid to the specific psychological mechanisms underlying depressive symptoms, such as cognitive fusion (CF) and ruminative response (RR) [31].

Our study aimed at exploring the potential relationships between cognitive fusion (CF), ruminative response (RR) and the presence of depressive signs and symptoms in a sample of university students. Our hypothesis was twofold: (a) CF and RR (for example, regarding past academic unwanted events, exams preparation, academic performance and current unwanted feelings) could be over-represented in university students compared to people of the same age who were involved in other work activities; (b) CF and RR might be the predictors of depressive signs and symptoms in university students.

## 2. Materials and Methods

### 2.1. Design and Participants

This was a cross-sectional study with a between-groups design. We recruited 181 young adult community volunteers between 1 June 2022 and 30 June 2022, employing an online procedure. Subjects who agreed to participate read the information on the protection of personal data before completing the online questionnaires and consented to the assessment. The data obtained were automatically transformed into codes to ensure participants’ anonymity at the very moment of completing the questionnaires. To be included in the study, participants had to be aged between 20 and 30 years, without any serious physical illness or current psychiatric disorder, including substances or alcohol use/abuse or suicidal ideation. We decided to include subjects aged at least 20 years in order to collect data on students who were engaged in academic activities (and related difficulties) for at least 1 year.

The Bioethics Committee of the University of Pisa approved the study (Prot. # 0077818/2022). All the methods in this study were carried out in accordance with the Declaration of Helsinki.

### 2.2. Questionnaires

We collected and analyzed data on demographic characteristics, including age, gender, marital status, employment status and educational level. Moreover, we utilized three self-assessment instruments described below, namely a questionnaire that measured the level of cognitive fusion (Cognitive Fusion Questionnaire-CFQ-7) [10], a questionnaire that collected depressive signs and symptoms (Depression-Zung Self-Assessment Scale) [32,33] and one exploring the ‘perseverative thinking modality’ (Perseverative Thinking Questionnaire-PTQ) [19].

#### 2.2.1. Cognitive Fusion Questionnaire-7 (CFQ-7)

CFQ-7 [10] consists of 7 items, which are rated on a Likert scale from ‘1’ to ‘7’. Higher CFQ-7 Total Scores indicate higher cognitive fusion (range: 7–49). Examples of CFQ items are ‘My thoughts cause me distress or emotional pain’ and ‘I tend to get very entangled in my thoughts’. Donati and colleagues (2021) [11] validated the Italian version of the scale, which demonstrated high internal consistency in clinical and non-clinical samples (Cronbach alpha coefficient = 0.88).

#### 2.2.2. Zung Self-Rating Depression Rating Scale (ZSDS) [32,33]

Zung SDS consists of 20 self-report items identified in factor analytic studies of depression. The items tap into psychological and physiological symptoms; ten explore negative experiences, such as ‘I feel down-hearted and blue’, and ten explore positive experiences and are reverse scored (e.g., ‘I eat as much as I used to’). Each item is rated according to how it applied within the past week using a 4-point Likert scale, ranging from ‘1′ (none or a little of the time) to ‘4’ (most or all of the time). Total scores range from ‘20′ to ‘80′. ZSDS has good internal consistency, with a split-half reliability of 0.73. ZSDS Total Scores are categorized as follows: range 20–31: ‘very low severity’ of depressive symptoms; range 32–43: ‘low severity’; range 44–55: ‘moderate severity’; range 56–67: ‘severe symptoms’; range 68–80: ‘very severe depressive symptoms’. Zung SDS demonstrated high internal consistency in clinical and non-clinical samples (Cronbach alpha coefficient in non-clinical samples = 0.85).

#### 2.2.3. Perseverative Thinking Questionnaire (PTQ) [19]

PTQ encompasses 15 items evaluating repetitive negative thinking (RNT) from a trans-diagnostic perspective in clinical and non-clinical samples. PTQ explores (1) the core characteristics of RNT, namely ‘repetitiveness’ (items 1, 6 and 11), ‘intrusiveness’ (items 2, 7 and 12) and the ‘difficulty in disengaging’ (items 3, 8 and 13); (2) the ‘perceived unproductiveness of RNT’ (items 4, 9 and 14); and (3) ‘RNT capturing mental resources’ (items 5, 10 and 15). Each item is scored according to a 5-point Likert scale from ‘0′ (‘never’) to ‘4′ (‘almost always’). A higher score in each dimension reflects a high level of the assumed process characteristic of the RNT considered. Validation studies reported good internal consistency of the scale (Cronbach alpha coefficient = 0.95) [19].

### 2.3. Statistical Analyses

The quantitative variables were described with means and/or medians and standard deviations and/or inter-quartile deviations. The qualitative variables were expressed with frequencies and percentages. The Shapiro–Wilk test was applied in order to verify whether the variables studied had normal distribution. The ANOVA test was used to compare the values of Gaussian quantitative variables. The Kruskal–Wallis test was performed for non-Gaussian variables. Any difference or association between nominal variables was computed using the Chi-square test or the Fisher exact test, depending on the frequencies detected. Student’s T-test was performed for continuous variables with normal distribution.

Correlations analyses were interpreted with the Pearson (r) or the Spearman (rs) correlation coefficients. Differences between groups were assessed with multivariate analysis of variance (MANOVA). The predictive validity of both cognitive fusion and perseverative thinking modality for the severity of depressive symptoms in US was assessed with a binary logistic regression analysis, with ZSDS scores < 44 vs. ≥44 as the dependent variable. A *p*-value < 0.05 was deemed significant. Analyses were carried out using IBM SPSS Statistics 20.

## 3. Results

### 3.1. Overview

The overall sample consisted of 181 young adults: 54 males (29.8%) and 127 females (70.2%). Two groups were compared according to the occupational role, namely university students (US) (*n* = 105/181; 58.0%) vs. non-university students (NUS) (*n* = 76/181; 42.0%). No statistically significant differences emerged in the gender distribution between US (36 M and 69 F) and NUS (18 M and 58 F) (χ^2^ = 0.084). The mean age of the overall sample was 25.30 ± 2.45 (range: 20–30). The mean age was not different between males and females (25.5 ± 2.3 vs. 25.2 ± 2.5, respectively; *p* = 0.270), but it was different between US and NUS (24.3 ± 2.4 vs. 26.6 ± 1.8, respectively; *p* = 0.0001). As a consequence, the MANOVA test comparing US vs. NUS was conducted using ‘Age’ as covariate. The demographic characteristics of US and NUS are summarized in Table 1.

US (*n* = 105) scored significantly higher than NUS (*n* = 76) in almost all domains of the scales, namely in the CFQ-7 Total Score (27.5 ± 9.4 vs. 24.4 ± 9.5; *p* = 0.040), ZSDS Total Score (41.1 ± 7.7 vs. 39.0 ± 7.3; *p* = 0.031), PTQ Total Score (26.1 ± 13.1 vs. 21.8 ± 13.9; *p* = 0.029), PTQ ‘Repetitiveness’ (5.3 ± 2.8 vs. 4.5 ± 2.9; *p* = 0.034), PTQ ‘Intrusiveness’ (5.8 ± 3.0 vs. 4.8 ± 3.1; *p* = 0.046) and PTQ ‘Repetitive Negative Thinking capturing mental resources’ (5.0 ± 3.1 vs. 4.0 ± 3.0; *p* = 0.013), in a MANOVA, corrected for age as covariate (Table 2). Then, we utilized a cut-off indicated by the ZSDS in order to dichotomize the severity of depressive signs and symptoms as ‘very low/low’ (ZSDS score < 44; *n* = 119) and ‘moderate/elevated/very elevated’ (ZSDS score ≥ 44; *n* = 62). No differences were found between NUS and US in the percentages of subjects who reached the ZSDS severity cut-off ≥44 (NUS = 27.6%; 21/76 vs. US = 39.0%; *n* = 41/105; χ^2^ = 0.110). Moreover, subjects who scored <44 (*n* = 119) vs. ≥44 (*n* = 62) in ZSDS did not differ in age distribution (25.2 ± 2.3 vs. 25.3 ± 2.6, respectively; Mann–Whitney test: *p* = 0.802). As expected, subjects with ZSDS severity score ≥ 44 scored significantly higher than subjects below the ZSDS threshold in all CFQ-7 and PTQ domains, as summarized in Table 3.

### 3.2. Correlation Analyses

Correlations analyses were interpreted with the Pearson r or the Spearman rs correlation coefficients where appropriate on PTQ, CFQ-7 and ZSDS scores. The total scores of the administered scales were significantly correlated in the overall sample, as well as in the two sub-samples (US and NUS). In the overall sample (*n* = 181), PTQ Total Score was positively correlated with CFQ-7 Total Score (r = 0.793; *p* < 0.001) and ZSDS Total Score (r = 0.709, *p* < 0.001), and CFQ-7 Total Score was positively correlated with ZSDS Total Score (r = 0.676, *p* < 0.001). In the NUS sub-sample (*n* = 76), PTQ Total Score was positively correlated with CFQ-7 Total Score (r = 0.788; *p* < 0.001) and ZSDS Total Score (r = 0.713, *p* < 0.001), and CFQ-7 Total Score was positively correlated with ZSDS Total Score (r = 0.771, *p* < 0.001). In the US sub-sample (*n* = 105), PTQ Total Score was positively correlated with CFQ-7 Total Score (r = 0.788; *p* < 0.001) and ZSDS Total Score (r = 0.697, *p* < 0.001), and CFQ-7 Total Score was positively correlated with ZSDS Total Score (r = 0.598, *p* < 0.001). The detailed results of correlation analyses are shown in Table 4a–e.

### 3.3. Binary Logistic Regression Analysis of Students with ZSDS Scores <44 vs. ≥44

We aimed at identifying which dimensions of RR and CF, as assessed with PTQ and CFQ-7, could be the predictors of a more severe depressive symptomatology in the US sample. We performed a binary logistic regression analysis of US, with ZSDS scores < 44 vs. ≥44 as the dependent variable, and PTQ Total Score and dimensions, CFQ-7 Total Score, age and gender (categorical) as the covariates. The only variable able to predict a more severe depressive symptomatology in students was the PTQ Total Score (OR = 1.44, 95% CI: 1.017–2.039; *p* = 0.040), as summarized in Table 5.

## 4. Discussion

The potential role of psychological inflexibility as the predictor of depressive symptoms in a general population sample of university students (US) was investigated in our study through the assessment of the levels of cognitive fusion (CF) and ruminative response style (RR).

Psychological inflexibility has been defined as a trans-diagnostic process, encompassing ‘an inability to effectively modify behavior in response to an immediate stressor or changing environmental demands’ [34]. In the short term, psychological inflexibility might be able to provide a ‘sense of relief’ from psychological suffering, with the adoption of a non-threatening way of regulating emotions [35]. Conversely, in the long term, psychological inflexibility might become maladaptive and might increase the risk of the onset of anxiety and depressive symptoms [36].

Studies conducted on university student samples demonstrated that higher levels of psychological inflexibility were correlated with higher risks of somatization, stress and generalized anxiety [37].

We aimed at exploring whether university students were a ‘special population’ of subjects more exposed to cognitive fusion (CF) and ruminative response (RR) and, as a consequence, to depressive symptoms than subjects of the same age who were not involved in academic activities.

We found that US experienced more depressive symptoms, more repetitive thinking modality, more intrusiveness of negative thinking and more mental resources drained by RNT, as well as higher levels of cognitive fusion than NUS.

Moreover, we found that the ‘perseverative thinking modality’ was the most relevant factor in predicting the severity of depressive signs and symptoms, making the severe depressive symptomatology more likely by 44%.

To our knowledge, this is the first study demonstrating that university students are a population more prone to experiencing CF and RR than subjects of the same age not involved in academic activities. Moreover, we found that these specific modalities of thinking might increase the risk of experiencing more severe depressive signs and symptoms in this population.

As far as we know, few studies explored the link between such psychological dimensions and depression in US, hypothesizing a specific role of ruminative response as a potential mediator in this path [29]. A recent study [11] considered CF among the variables influencing subjective well-being in a sample of university students; another study validated the European Portuguese version of the acceptance and action questionnaire for university students, exploring the context-specific psychological inflexibility in two different samples of university students [38].

Conversely, a large number of studies considered other practical or social variables. For example, according to a systematic review of the occurrence of depressive episodes in USA college students, nearly one-third of the sample (30.6%) described significant depressive symptoms [5]. A study of 13.984 university students from eight countries reported a prevalence of 21% for a full-blown depressive episode [6]. In a more recent study of 1074 university students with a mean age of 21 years, a prevalence of 23.6% for anxiety and 18.4% for depressive symptoms was found [39]. A recent study of 1102 Italian university students (mean age 22 years ± 3.28) found moderate depressive symptoms in 22% and severe depressive symptoms in 12% of respondents [40]. However, taken as a whole, these studies pointed out the role of variables other than CF and RR, such as physical distance from significant others, the economic burden of university studies, or new environmental or interpersonal and social demands, belonging to a difficult ‘role transition’ [41].

According to these observations, the transition from school to university can be experienced by most university students as challenging mainly because of the increase in workload and academic standard [42], the occurrence of interpersonal deficit and loneliness [43], the exacerbation of homesickness [44,45] or the occurrence of difficulties in future orientation and/or in finding academic intrinsic motivations [41], leading to academic under-performance or to withdrawal from university [44].

Our study, starting from a different perspective, aimed at exploring the theoretical premises of the acceptance and commitment therapy (ACT), namely the excessive control attributed to thought and to the lack of action as specific ‘intensifiers’ of depressed mood [10].

This theoretical premise seems to be confirmed by our findings, considering that US were more affected by depressive symptoms than NUS, that cognitive fusion and ruminative response were over-represented in US compared to NUS and that PTQ was able to predict the severity of depressive symptoms in US, raising the odds of severe symptoms to around 44%.

We could hypothesize that the focus on negative internal emotional states, characteristic of rumination, might be more protracted in US than in subjects involved in other types of activities and might significantly contribute to the maintenance of depressed mood because of the tendency to consider academic results as their major concern, the pressure to achieve good grades, a negative perception of workload and less certainty in post-academic perspectives [46]. We could also hypothesize that our findings could be at least partially influenced by the COVID-19 pandemic, which modified US lifestyle in several ways, including social distancing restrictions, the impossibility to access universities and societal changes in technology usage [26,27]. Thus, we collected the data in June 2022, when the lockdown in Italy was terminated, but US were scheduled for the entire academic year 2021–2022 as a digital learning experience, with no face-to-face interactions with peers and teachers.

We could also speculate that the interpersonal deficit derived from the COVID-19 pandemic might have had a more significant influence on US than on subjects involved in work activities. Interpersonal deficit is one of the focuses related to the onset of depressive episodes, according to the interpersonal psychotherapy (IPT) model proposed by Weissman [47]. Following the IPT paradigm, interpersonal dysfunctions might be the precursors, prodromes or sequalae of a depressive episode, especially during late adolescence and early adulthood [48].

During the COVID-19 pandemic, university students became more isolated from the normalizing influence of their peers, with significant changes in their activity routines. The ‘social zeitgeber’ hypothesis posited that unstable or disrupted daily routines might lead to circadian rhythm instability and, in vulnerable individuals, to mood instability, sleep disorders and mood disorders [49,50]. According to this model, psychosocial factors interact with biology to create a pathway to psychopathology, where disruption to the bio-psychosocial rhythms plays a key role. For example, a recent study found that psychological inflexibility was able to mediate the relationship between sleep rhythm disruption in college students and the onset of depressive symptoms [51]. The overall change in daily routines might become more evident in US who followed their lessons from a remote setting and with no direct interaction with peers than in subjects who continued to have their psychosocial rhythms regulated by their work activities, even in a ‘smart working’ modality.

Our study had several limitations. The study had a cross-sectional design, which did not allow observing the selected variables at different time intervals but only in a specific time frame; this entailed a greater difficulty in interpreting the causal relationships between variables. The questionnaires administered were all self-reports; therefore, they might have been subject to recall bias from participants. The sample size was small. A further limitation was the absence of a clinical comparison group, which would have made it possible to evaluate the differences and/or similarities in the scores of cognitive fusion and rumination between clinical and non-clinical samples.

## 5. Conclusions

The study, albeit with limitations, highlighted for the first time, to the best of our knowledge, the role of cognitive fusion processes and ruminative response style in predicting vulnerability to depressive spectrum symptoms and their severity in a sample of university students compared to non-university students of the same age. We believe that US should be considered as a ‘special population’ at risk of depression, especially when a ruminative style of thinking is present, and that they should benefit from specific programs of psychoeducational support or psychotherapies specifically targeted at cognitive fusion.

## Figures and Tables

**Table 1:** Demographic characteristics of the sample of US (*n* = 105) vs. NUS (*n* = 76).

	US (*n* = 105)	NUS (*n* = 76)
**Age (mean/SD) ^**	26.6 ± 1.8	24.3 ± 2.4
Status * (*n*/%)		
*Single*	91 (86.6)	55 (72.3)
*In a relationship*	7 (6.6)	1 (1.3)
*Married*	7 (6.6)	20 (26.3)
Work Status * (*n*/%)		
*Unemployed*	-	9 (11.8)
*Student*	105 (100)	-
*Soldier*	-	1 (1.3)
*Worker/Workman*	-	5 (6.5)
*Employee*	-	37 (48.6)
*Freelance*	-	20 (26.3)
*Manager*	-	1 (1.3)
*Trader*	-	3 (3.9)
School Level ** (*n*/%)		
*Middle School*	-	1 (1.3)
*High School*	33 (31.4)	15 (19.7)
*Degree*	67 (63.8)	48 (63.1)
*Master*	5 (4.7)	12 (15.7)

^ *p* = 0.0001  * χ^2^ = 0.0001  ** χ^2^ = 0.025.

**Table 2:** PTQ, CFQ-7 and ZSDS scores in university students (US) vs. non-university students (NUS) *.

	NUS (*n* = 76) Mean/SD	US (*n* = 105) Mean/SD	*p*
PTQ Total Score	21.8 ± 13.9	26.1 ± 13.1	0.029
Repetitiveness	4.5 ± 2.9	5.3 ± 2.8	0.034
Intrusiveness of RNT	4.8 ± 3.1	5.8 ± 3.0	0.046
Difficulty in disengaging from RNT	4.2 ± 2.9	5.0 ± 3.0	0.100
Perceived unproductiveness of RNT	4.3 ± 2.7	4.8 ± 2.7	0.156
RNT capturing mental resources	4.0 ± 3.0	5.0 ± 3.1	0.013
CFQ-7 Total Score	24.4 ± 9.5	27.5 ± 9.4	0.040
ZSDS Total Score	39.0 ± 7.3	41.1 ± 7.7	0.031
ZSDS Threshold			χ^2^
ZSDS < 44	55 (72.4)	64 (61.0)	0.110
ZSDS ≥ 44	21 (27.6)	41 (39.0)	

* Multivariate analysis of variance (MANOVA) with ‘Age’ as covariate.

**Table 3:** PTQ and CFQ-7 scores in subjects with ZSDS score < 44 (*n* = 119) vs. subjects with ZSDS score ≥ 44 (*n* = 62).

	ZSDS < 44 (*n* = 119) Mean/SD	ZSDS ≥ 44 (*n* = 62) Mean/SD	*p*
PTQ Total Score	18.0 ± 10.1	36.3 ± 11.1	0.0001 a
Repetitiveness	3.7 ± 2.3	7.2 ± 2.3	0.0001 b
Intrusiveness of RNT	4.2 ± 2.6	7.5 ± 2.6	0.0001 a
Difficulty in disengaging from RNT	3.3 ± 2.3	7.2 ± 2.4	0.0001 b
Perceived unproductiveness of RNT	3.5 ± 2.1	6.8 ± 2.3	0.0001 b
RNT capturing mental resources	3.1 ± 2.3	7.4 ± 2.6	0.0001 a
CFQ-7 Total Score	22.3 ± 8.5	33.8 ± 6.6	0.0001 a

a, Student’s *T*-test for independent samples; b, ANOVA for independent samples.

**Table 4:** Correlation analyses between PTQ, CFQ-7 and ZSDS total scores.

(a) Overall Sample (*n* = 181).							
	CFQ-7 Total ^a^	ZSDS Total ^a^	PTQ Repetitiveness ^a^	PTQ Intrusiveness ^a^	PTQ Difficulty in Disengaging ^b^	PTQ Unproductiveness ^b^	PTQ Mental Resources ^a^
PTQ Total	0.793 **	0.709 **	0.935 **	0.889 **	0.946 **	0.851 **	0.915 **
CFQ-7 Total	-	0.676 **	0.758 **	0.689 **	0.736 **	0.712 **	0.710 **
ZSDS Total	-	-	0.663 **	0.557 **	0.639 **	0.675 **	0.687 **
PTQ Repetitiveness	-	-	-	0.782 **	0.891 **	0.779 **	0.828 **
PTQ Intrusiveness	-	-	-	-	0.848 **	0.678 **	0.730 **
PTQ Difficulty in dis.	-	-	-	-	-	0.739 **	0.836 **
PTQ Unproductiveness	-	-	-	-	-	-	0.767 **
**(b) NUS (*n* = 76).**							
	**CFQ-7 Total ^a^**	**ZSDS Total ^a^**	**PTQ Repetitiveness ^a^**	**PTQ Intrusiveness ^a^**	**PTQ Difficulty in Disengaging ^b^**	**PTQ Unproductiveness ^b^**	**PTQ Mental Resources ^a^**
PTQ Total	0.788 **	0.713 **	0.942 **	0.907 **	0.944 **	0.917 **	0.936 **
CFQ-7 Total	-	0.771 **	0.725 **	0.654 **	0.682 **	0.747 **	0.719 **
ZSDS Total	-	-	0.619 **	0.598 **	0.590 **	0.653 **	0.667 **
PTQ Repetitiveness	-	-	-	0.782 **	0.884 **	0.842 **	0.888 **
PTQ Intrusiveness	-	-	-	-	0.849 **	0.796 **	0.787 **
PTQ Difficulty in dis.	-	-	-	-	-	0.809 **	0.857 **
PTQ Unproductiveness	-	-	-	-	-	-	0.834 **
**(c) US (*n* = 105).**							
	**CFQ-7 Total ^a^**	**ZSDS Total ^a^**	**PTQ Repetitiveness ^a^**	**PTQ Intrusiveness ^a^**	**PTQ Difficulty in Disengaging ^b^**	**PTQ Unproductiveness ^b^**	**PTQ Mental Resources ^a^**
PTQ Total	0.788 **	0.697 **	0.930 **	0.854 **	0.943 **	0.797 **	0.882 **
CFQ-7 Total	-	0.598 **	0.762 **	0.673 **	0.741 **	0.660 **	0.651 **
ZSDS Total	-	-	0.672 **	0.483 **	0.635 **	0.662 **	0.665 **
PTQ Repetitiveness	-	-	-	0.740 **	0.881 **	0.721 **	0.779 **
PTQ Intrusiveness	-	-	-	-	0.824 **	0.562 **	0.664 **
PTQ Difficulty in dis.	-	-	-	-	-	0.646 **	0.804 **
PTQ Unproductiveness	-	-	-	-	-	-	0.664 **
**(d) ZSDS < 44 (*n* = 119).**							
	**CFQ-7 Total ^a^**	**ZSDS Total ^a^**	**PTQ Repetitiveness ^a^**	**PTQ Intrusiveness ^a^**	**PTQ Difficulty in Disengaging ^b^**	**PTQ Unproductiveness ^b^**	**PTQ Mental Resources ^a^**
PTQ Total	0.692 **	0.480 **	0.887 **	0.824 **	0.910 **	0.765 **	0.837 **
CFQ-7 Total	-	0.540 **	0.645 **	0.531 **	0.591 **	0.628 **	0.524 **
ZSDS Total	-	-	0.454 **	0.294 **	0.358 **	0.501 **	0.412 **
PTQ Repetitiveness	-	-	-	0.629 **	0.817 **	0.627 **	0.720 **
PTQ Intrusiveness	-	-	-	-	0.760 **	0.506 **	0.559 **
PTQ Difficulty in dis.	-	-	-	-	-	0.581 **	0.692 **
PTQ Unproductiveness	-	-	-	-	-	-	0.614 **
**(e) ZSDS ≥ 44 (*n* = 62).**							
	**CFQ-7 Total ^a^**	**ZSDS Total ^a^**	**PTQ Repetitiveness ^a^**	**PTQ Intrusiveness ^a^**	**PTQ Difficulty in Disengaging ^b^**	**PTQ Unproductiveness ^b^**	**PTQ Mental Resources ^a^**
PTQ Total	0.676 **	0.291 *	0.934 **	0.856 **	0.923 **	0.784 **	0.891 **
CFQ-7 Total	-	0.169	0.609 **	0.592 **	0.611 **	0.525 **	0.639 **
ZSDS Total	-	-	0.252*	0.210	0.146	0.324 *	0.282 *
PTQ Repetitiveness	-	-	-	0.764 **	0.878 **	0.700 **	0.801 **
PTQ Intrusiveness	-	-	-	-	0.808 **	0.537 **	0.665 **
PTQ Difficulty in dis.	-	-	-	-	-	0.614 **	0.775 **
PTQ Unproductiveness	-	-	-	-	-	-	0.735 **

** *p* < 0.01 (two-tailed); * *p* < 0.05; a = Pearson correlation; b = Rho Spearman correlation.

**Table 5:** Binary logistic regression analysis of university students (*n* = 105) with ZSDS scores <44 vs. ≥44.

	B	E.S.	Wald	df	Sig.	Exp(B)	95% CI for EXP(B)	
							Inf	Sup
Age	0.076	0.119	0.407	1	0.523	1.079	0.855	1.361
Gender	0.152	0.667	0.052	1	0.820	1.164	0.315	4.307
CFQ-7 Total Score	−0.030	0.059	0.253	1	0.615	0.971	0.865	1.090
PTQ Total Score	0.365	0.178	4.213	1	0.040	1.440	1.017	2.039
‘Repetitiveness’	−0.468	0.323	2.095	1	0.148	0.626	0.332	1.180
‘Intrusiveness’	−0.442	0.246	3.214	1	0.073	0.643	0.397	1.042
‘Difficulty in disengaging from RNT’	0.069	0.364	0.036	1	0.849	1.071	0.525	2.186
‘Perceived unproductiveness of RNT’	0.029	0.246	0.014	1	0.905	1.030	0.636	1.666

## Data Availability

Data are unavailable due to privacy and ethical restrictions.
